# Combined minimally invasive resection of thoracic neurogenic dumbbell tumors: A European case series

**DOI:** 10.1111/1759-7714.14122

**Published:** 2021-08-23

**Authors:** Oliver J. Harrison, Adnan Bakir, Martin H. Chamberlain, Ali Nader‐Sepahi, Khalid M. Amer

**Affiliations:** ^1^ Department of Thoracic Surgery University Hospital Southampton Southampton UK; ^2^ Faculty of Medicine University of Southampton Southampton UK; ^3^ Department of Neurosurgery University Hospital Southampton Southampton UK

**Keywords:** minimally invasive surgery, neurogenic thoracic tumors, surgical oncology, video‐assisted thoracoscopic surgery

## Abstract

**Background:**

Paraspinal tumors are rare neoplasms arising from neurogenic elements of the posterior mediastinum and surgical resection can be challenging. Here, we demonstrate feasibility and outcomes from the first European case series of combined laminectomy and video‐assisted thoracoscopic surgery (VATS) resection of thoracic neurogenic dumbbell tumors.

**Methods:**

A retrospective review of all combined thoracic dumbbell tumor resections performed at our institution between March 2015 to February 2019 was undertaken. Outcomes included operative time, blood loss, length of stay and recurrence rate. Statistical analysis was performed with SPSS statistics (v26). Values are given as mean ± standard deviation and median ± interquartile range.

**Results:**

Seven patients were included in the case series and there were no major complications or mortality. Mean tumor size and operative time were 66 (± 35) mm and 171 (± 63) min, respectively. Median blood loss and length of stay were 40 (± 70) ml and four (± 3) days, respectively. One patient required conversion to thoracotomy to remove a tumor of 135 mm in maximal dimension. Histology in all seven cases confirmed schwannoma. There was no disease recurrence at a maximum follow‐up of 54 months.

**Conclusions:**

Our experience demonstrates favorable operative times, minimal blood loss and short length of stay when dealing with relatively large tumors compared to previous reports. Thoracotomy may be required for tumors exceeding 90 mm and chest drain removal on the operative day can facilitate early mobility and discharge. We advocate a combined, minimally invasive laminectomy and VATS resection as the gold‐standard approach for thoracic neurogenic dumbbell tumors.

## Graphical abstract

Paraspinal tumors are rare and surgical resection can be challenging. Optimal surgical strategy is contentious. We demonstrate outcomes from the first European case series of combined microlaminectomy and video‐assisted thoracoscopic surgery. Our experience demonstrates favorable operative times, minimal blood loss and short length of stay when dealing with relatively large tumors compared to previous reports. Removing the chest drain on the operative day facilitates early discharge. We advocate a combined minimally invasive approach as the gold‐standard for paraspinal tumor resection.

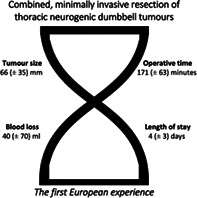

## INTRODUCTION

Neurogenic tumors of the posterior mediastinum are associated with an intraspinal component in approximately 10% of cases.^1^ Histological subtypes are based on the origin of the tumor which may be nerve sheath (schwannomas and neurofibromas), autonomic ganglion (ganglioneuromas and neuroblastomas) or paraganglionic (paragangliomas and pheochromocytomas).^2^ In adults, the vast majority (>95%) are benign.^3^ Relative narrowing of the tumor in the intervertebral foramen gives the characteristic “dumbbell” appearance. First described as “hourglass” shaped in 1929 by Heuer, the term “dumbbell” was more recently coined by Love and Dodge in 1952.^4^, ^5^

The combined approach to neurogenic tumor excision with both thoracic and neurosurgical components was described over 40 years ago.^1^, ^6^ In the absence of neurological symptoms, diagnosis of a dumbbell tumor relied on plain x‐ray films, which often demonstrated erosion of the vertebral pedicle or enlargement of the intervertebral foramen.^1^ Complications of an isolated thoracic approach were recognized at this early stage. Excessive traction on an undiagnosed spinal extension can cause spinal cord injury and bleeding may be impossible to control. With the advent of modern imaging methods, accurate preoperative diagnosis and planning of surgical approach is now possible. Refinement of a single‐stage procedure with minimally invasive microneurosurgical and video‐assisted thoracoscopic surgery (VATS) techniques was first described by Vallières et al. in 1995.^7^ This approach facilitates a rapid recovery with reduced postoperative pain and return to preoperative function.^2^, ^8^, ^9^, ^10^, ^11^, ^12^, ^13^ There is a general consensus that removing the tumor anteriorly without prior mobilization from the spinal cord by laminectomy is unsafe.^14^ Here, we present the results from the first European case series of combined laminectomy and VATS resection for thoracic dumbbell paraspinal neurogenic tumors.

## METHODS

### Patient selection, outcome measures and statistical analysis

**FIGURE 1:**
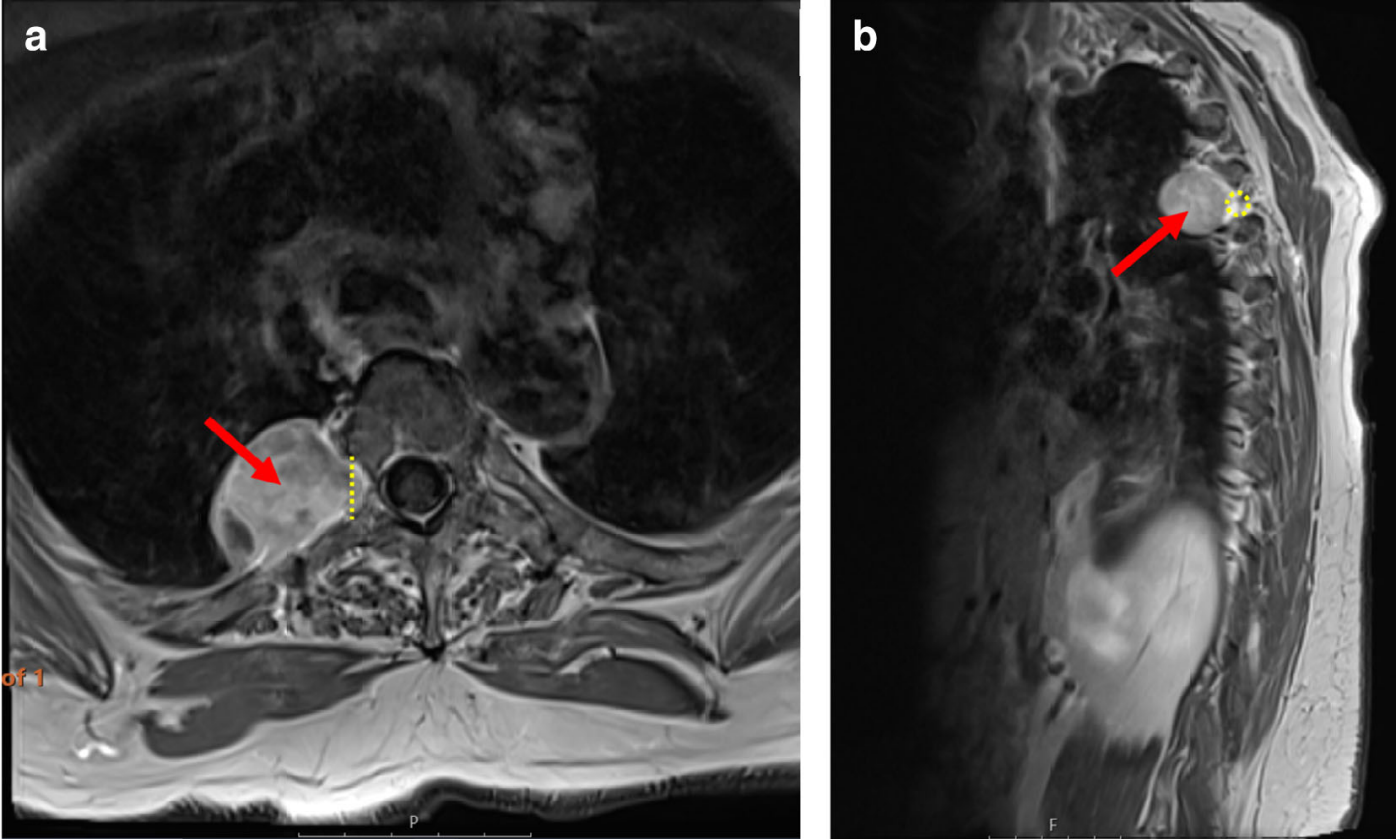
Preoperative T1 weighted magnetic resonance imaging (MRI) of Patient 3 demonstrating a paraspinal dumbbell tumor in the right hemithorax at the T4/5 level (red arrow) extending into the intervertebral foramen (yellow dotted line and circle). (a) axial section; (b) sagittal section

Written informed consent was obtained for intraoperative photography and use of this in the current publication. A retrospective review of case notes for all patients undergoing combined laminectomy and video‐assisted thoracoscopic surgery (VATS) resection of thoracic neurogenic dumbbell tumors between March 2015 and February 2019 at University Hospital Southampton (UK) was undertaken. All patients underwent joint thoracic and neurosurgical multidisciplinary review, which included whole spine MRI and chest CT imaging (Figure 1). Procedures were performed by a single neurosurgeon and two thoracic surgeons. Outcomes included operative time, blood loss, postoperative complications, length of stay and recurrence. Mean values are given ± standard deviation. Median values are given with interquartile range. Correlations were calculated using the Pearson coefficient. *p* < 0.05 was taken to be statistically significant. Statistical analysis was performed using IBM SPSS statistics (version 26).

### Surgical approach and postoperative care

**FIGURE 2:**
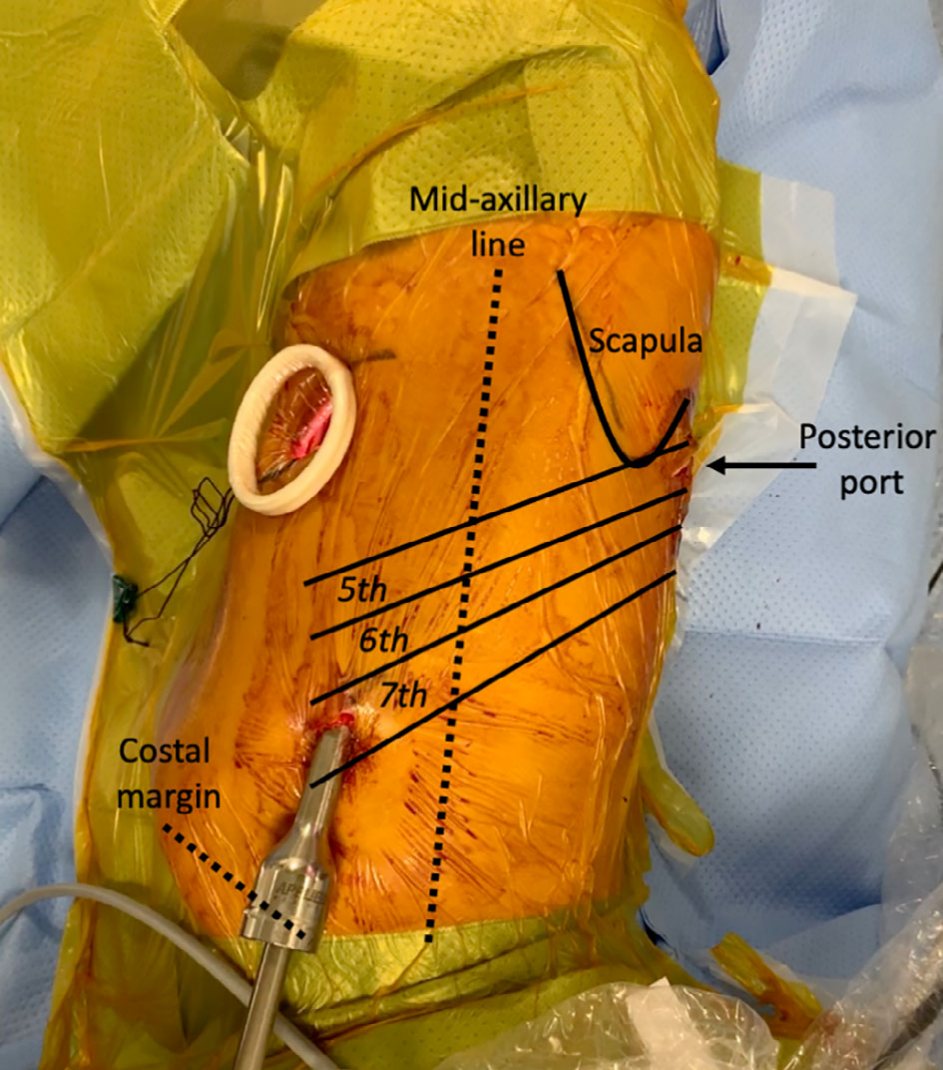
Standard 3‐port video‐assisted thoracoscopic surgery (VATS) port placement for the thoracic component of the dumbbell tumor resection (top, cranial; bottom, caudal; left, anterior; right, posterior)

**FIGURE 3:**
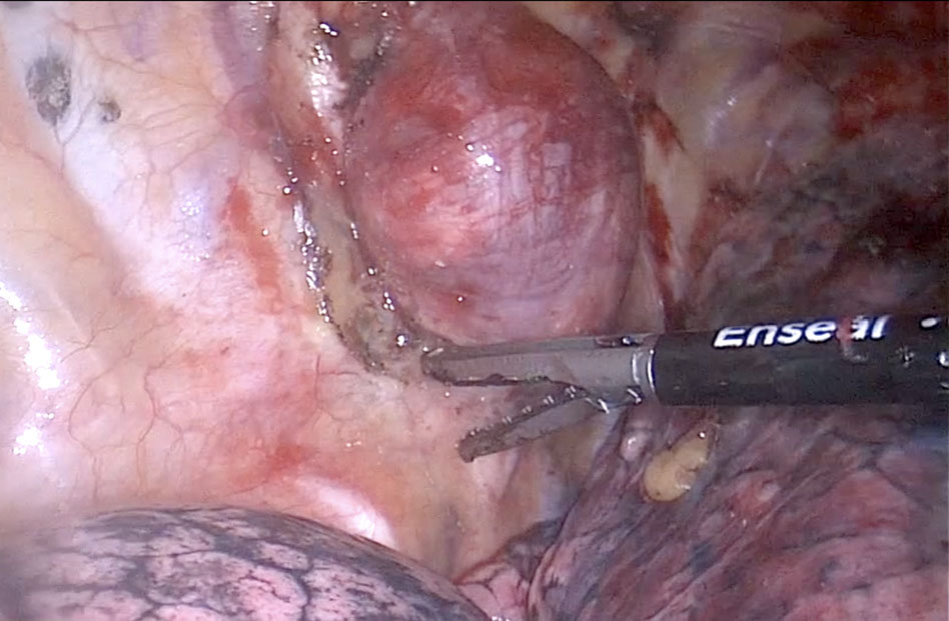
Thoracoscopic view of the paraspinal tumor. The parietal pleura surrounding the tumor is mobilized

The involved vertebral level of the tumor is marked on the skin of the patient's back with intraoperative fluoroscopy. Under general anesthesia, the patient is intubated with a double‐lumen endotracheal tube and placed in the prone position. A vertical midline incision is made to expose the hemi‐laminae, facet joint and the relevant transverse processes unilaterally at the selected levels. The degree of bony resection is tailored to the extent of the tumor medially in the spinal canal and laterally outside the foramen. Under the operating microscope, enough bone is removed to expose the tumor and its relationship to the neural elements (spinal cord and the exiting nerve) as well as the dural sleeve. The tumor is completely mobilized away from the spinal cord and the adjacent nerve roots. After removing this component of the tumor and dividing the fascicle of the nerve giving rise to the tumor, watertight closure of the dura is achieved and the suture line is covered with Tissue Patch Dural (Tissuemed Ltd, UK). The remaining tumor with the distal nerve stump is pushed into the chest cavity via the enlarged foramen. The tumor bed and the neural foramen is inspected for haemostasias. After the posterior incision is closed, the patient is placed in the lateral decubitus position for VATS. The operating table is flexed at the torso to open the intercostal spaces. Port positioning is approached with surgeon anterior to the patient, and triangulation is designed for three‐port VATS (Figure 2). Carbon dioxide (CO_2_) is used to insufflate the chest at a maximum pressure of 10 mmHg and a flow of 1 liter per min. CO_2_ is avoided if the dura has been opened. We commonly use an ENSEAL bipolar energy device (Johnson & Johnson, USA), which is particularly useful in the setting of paraspinal tumor resection because the articulating head aligns well with the interface between tumor and chest wall. Similarly, bipolar delivery minimizes energy spread outside the jaws of the device reducing potential damage to the intercostal nerve and vessels. The ENSEAL is used to dissect around the tumor, starting by lifting the pleura from a 1 cm distance (Figure 3). If the tumor is stuck to the intercostal nerve then the latter is removed with the tumor. The specimen is removed within an Anchor endoscopic retrieval bag (Conmed, USA). A single 28F chest tube is inserted via the inferior port and connected to a digital Thopaz drainage device (Medela, UK Ltd, UK). Chest tubes are removed 2 hours postoperatively if full lung expansion is confirmed on chest x‐ray and there is no recorded air leak or ongoing blood loss. Patients are then transferred to the neurological high dependency unit for care under the neurosurgical team until discharge.

## RESULTS

### Patient characteristics

A summary of the patient characteristics is given in Table 1. Over the 4‐year study period, seven patients underwent combined resections. Patient 1 represents the first combined case performed at our institution (March 2015). Of note, a further seven thoracic neurogenic dumbbell tumors were resected during the study period but these were either non‐combined procedures or performed via a thoracic approach only. Mean age was 64 (± 14) years and four of seven patients were male.

**TABLE 1:** Patient characteristics

Case ID	Age	Gender	Tumor laterality and level	Tumor size (T × AP × CC; mm)	Tumor histology	Operative time (min)	Blood loss (ml)	Complications (intra‐ or postoperative)	Length of stay (days)	Length of follow‐up (months)	Recurrence
1	74	Female	Right; T10/11	90 × 70 × 60	Schwannoma	255	40	Neuropathic pain controlled with medication	6	44	No
2	49	Male	Right; T5/6	135 × 110 × 95	Schwannoma	240	720	Converted to thoracotomy; chest drain reinsertion postoperative day 3	4	54	No
3	78	Female	Right; T4/5	40 × 25 × 20	Schwannoma	170	20	Nil	13	5	No
4	72	Male	Left; T10/11	60 × 45 × 20	Schwannoma	200	100	Nil	6	13	No
5	58	Male	Left; T8/9	37 × 30 × 25	Schwannoma	105	50	Nil	2	4	No
6	77	Female	Left; T3/4	45 × 25 × 25	Schwannoma	100	40	Nil	3	15	No
7	42	Male	Right; T12/L1	55 × 35 × 25	Schwannoma	125	30	Nil	4	9	No

Abbreviations: AP, anteroposterior; CC, craniocaudal; T, transverse.

### Operative management

All patients successfully underwent combined, single stage resection. All but one procedure was completed entirely by minimally invasive surgery. On this occasion, a thoracotomy was required to deliver the tumor, which was 135 mm in maximal dimension. The tumor was also adherent to the lung which required wedge resection for its complete removal. Mean operative time was 171 (± 63) min and there was a significant decrease in operative time over the course of the study period (*p* = 0.012; *r* = −0.866). Median estimated blood loss was 40 (± 70) ml.

### Tumor characteristics

Four tumors were located on the right and tumor level varied from T3/4 to T12/L1. Mean tumor size was 66 (± 35) mm. Histology confirmed all seven tumors were schwannomas.

### Hospital stay, complications and follow‐up

Median length of stay was 4 (± 3) days. The patient converted from VATS to thoracotomy required chest drain reinsertion on postoperative day 3 for subcutaneous emphysema. Patient 3 had a prolonged hospital stay due to a lower respiratory tract infection requiring intravenous antibiotics. Patient 1 experienced prolonged neuropathic chest pain requiring regular analgesia to control. There was no mortality. Mean duration of follow‐up was 27 (± 17) months at which time there were no recurrences on repeat MRI imaging. Patient 3 was lost to follow‐up.

## DISCUSSION

Paraspinal neurogenic tumors are rare neoplasms arising from neurogenic elements of the posterior mediastinum. Schwannomas constitute 90% of all paraspinal tumors, which is consistent with the findings in our study.^15^ Surgical removal of these tumors with mediastinal, neuroforaminal and intraspinal components can often be challenging. A recurrence risk of 5% at a mean interval of 1.7 years necessitates complete resection, which can be reliably achieved with a laminectomy and VATS resection.^16^ We present the first European case series of combined, minimally invasive neurosurgical and thoracic resection of dumbbell paraspinal tumors in a single‐stage operation. Although this was a relatively small cohort, we have demonstrated favorable outcomes in both hemithorax at multiple spinal levels with no recurrence supporting the technique as safe and feasible. Where tumors exceed 90 mm in maximal diameter, we advocate considering thoracotomy as the standard approach to avoid risk of prolonged operative time and need for conversion. There were no complications attributed to the use of CO_2_ in our series. However, there is a theoretical risk of gas leaking into the theca, especially at pressures exceeding 10 mmHg. The neurosurgeon always confirms the theca to be hermetically sealed and an absence of CSF leak before the VATS procedure commences.

The perceived advantage of a staged procedure, removing the intraspinal component on a separate occasion from the intrathoracic component, is mainly logistical. Coordination between neurosurgical and the thoracic surgical teams is minimal and both teams are free to operate within their own theater environment, with their own equipment, on their own schedule. Nonetheless, there are inherent dangers with this approach. Historically, in our institution the neurosurgical component was performed first followed 3 months later by the thoracic component. The main concern of the thoracic surgeon lies around dissection close to the intervertebral foramen, where it is presumed clearance has taken place during the initial neurosurgical procedure. However, after 3 months it can be very difficult to make judgment about the nature of the tissue in that area. A differentiation between postoperative fibrosis and residual tumor can be impossible. This risk is mitigated in the single‐stage approach, where tissue planes are clear and frequently once the pleura is divided, the tumor will simply fall away into the chest. The neurosurgeon can always be called into the thoracic part of the procedure to advise on completeness of resection. In our experience, we have not needed to revisit the posterior approach, but this remains an option should the need arise.

Only a handful of other studies have previously reported outcomes of combined VATS and laminectomy resection (Table 2). Nam et al. reported a case of paraspinal hemangioma resection complicated by bleeding and advocated preoperative embolization in such rare cases.^11^ Similarly, the invasiveness of the combined approach with VATS is questioned in light of prolonged operative times in their series (331 min). However, our mean operative time of under 3 h suggests prolonged operative time need not be a disadvantage and operative time decreases with experience. Chen et al. included analysis of four laminectomy and thoracotomy cases. The mean size of the tumors in this group was 91 mm (range 8–12).^13^ Although we were able to remove a tumor of 90 mm via a VATS approach, these previous data support our recommendation to strongly consider thoracotomy when dealing with tumors in excess of 90 mm.

**TABLE 2:** Outcomes reported in previous publications compared to the present study

Study	Year	Participant number	Tumor size (mm)	Operative time (min)	Estimated blood loss (ml)	Length of stay (days)
Vallières et al.^7^	1995	4	53	NR	NR	NR
Barrenechea et al.^2^	2006	3	51	328	483	4
Nam et al.^11^	2017	7	NR	331	348	NR
Li et al.^12^	2018	20	65	305	619	11
Chen et al.^13^	2019	10	50	244^a^	360^a^	4^a^
Harrison et al. (present study)	2021	7	66	171	40	4

*Note*: Average values are given as reported by the respective studies. NR, value not reported.

^a^ Value calculated with 10 additional cases which were not laminectomy and VATS (isolated VATS, thoracotomy plus laminectomy, supraclavicular approach and supraclavicular approach plus thoracotomy).

Other groups advocate a posterior (spinal only) approach as standard for thoracic dumbbell tumors. Li et al. reported favorable operative time (222 min), blood loss (400 ml), length of stay (8 days) and reduced pulmonary complications versus a combined approach.^12^ Rong et al. reported a series of 14 patients undergoing posterolateral resections without instrumentation.^17^ Mean operative time and estimated blood loss were comparable with Li et al. (272 min and 529 ml, respectively); however, one patient had subtotal resection and recurrence of disease. Zairi et al. reported a series of five dumbbell tumors with mean maximal dimension of 46 mm resected by a posterior‐only approach.^18^ Mean operative time was 219 min, mean blood loss was 230 ml and mean length of stay was 3.6 days. One patient had subtotal resection due to the tumor being adherent to the pleura and diaphragm, but no recurrence was reported after 4 years follow‐up. Ando et al. reported a mean operative time of 320 min and blood loss of 540 ml in a series of 16 patients undergoing a posterior only approach.^19^ Pleural injury complicated one case and required repair. Whilst the authors advocate the posterior approach, they suggest that any involvement of the intercostal artery should necessitate a combined thoracoscopic procedure to minimize risk of spinal cord bleeding. More recently, a combined approach using robot‐assisted thoracic surgery allowed the patient to remain in the prone position for the entire operation and this may represent a future direction for minimally invasive combined resection of thoracic neurogenic dumbbell tumors.^20^

Avoidance of chest drain placement is cited as a major advantage of the posterior approach. However, prolonged chest drainage is not a necessity of the combined approach. The evolving practice in our institution is to remove the chest drain in recovery (in the absence of air leak and bleeding) before the patient is transferred to the neurological high dependency unit. Consequently, risks of pulmonary complications (intercostal neuralgia, pulmonary atelectasis and pneumonia) are minimized and patients are free to mobilize early. This is reflected in our relatively short length of stay of four days. Furthermore, the avoidance of spinal instrumentation is favorable for early mobilization and reducing risk of deep wound infection, chronic back pain, deformity and instability.^7^, ^8^, ^14^ The posterior approach may be considered in units without thoracic surgery services where there is no suspicion of vascular involvement or underlying hemangioma. However, we would advocate routine referral to a unit with both neurosurgical and thoracic surgical services to facilitate combined, minimally invasive resection. This should be via thoracotomy if the tumor exceeds 90 mm in maximal dimension and may involve a supraclavicular approach if the tumor is at a high thoracic or low cervical level.^21^ Ideally, all patients should be seen by both a neurosurgeon and a thoracic surgeon to discuss the operation and consent in a joint clinic.

This case series has a number of limitations. Our cohort is small, mainly because these cases are rare and infrequently encountered. Prospective review is ongoing to increase our case numbers and experience, which we hope will strengthen our understanding of the surgical management of this disease and the conclusions of the study.

In conclusion, thoracic dumbbell paraspinal neurogenic tumors are rare and infrequently encountered. We advocate a combined, minimally invasive approach with a multidisciplinary thoracic and neurosurgical team as the gold‐standard approach to resection. Our experience demonstrates favorable operative times, minimal blood loss and short length of stay when dealing with relatively large tumors compared to previous reports. With the intent to potentially expedite recovery and minimize pulmonary complications, chest drain removal on POD 0 appears feasible and safe in most patients.
